# Human-induced fire regime shifts during 19^th^ century industrialization: A robust fire regime reconstruction using northern Polish lake sediments

**DOI:** 10.1371/journal.pone.0222011

**Published:** 2019-09-16

**Authors:** Elisabeth Dietze, Dariusz Brykała, Laura T. Schreuder, Krzysztof Jażdżewski, Olivier Blarquez, Achim Brauer, Michael Dietze, Milena Obremska, Florian Ott, Anna Pieńczewska, Stefan Schouten, Ellen C. Hopmans, Michał Słowiński

**Affiliations:** 1 Alfred-Wegener-Institute Helmholtz Center for Polar and Marine Research, Research Unit Potsdam, Polar Terrestrial Environmental Systems, Potsdam, Germany; 2 GFZ German Research Centre for Geosciences, Section Climate Dynamics and Landscape Evolution, Potsdam, Germany; 3 Polish Academy of Sciences, Institute of Geography and Spatial Organization, Toruń, Poland; 4 Royal Netherlands Institute for Sea Research, Department of Marine Microbiology and Biogeochemistry, and Utrecht University, Texel, The Netherlands; 5 Museum of the Kościerzyna Land, Kościerzyna, Poland; 6 Département de Géographie, Université de Montréal, Montréal, Québec, Canada; 7 GFZ German Research Centre for Geosciences, Section Geomorphology, Potsdam, Germany; 8 Polish Academy of Sciences, Institute of Geological Sciences, Warsaw, Poland; 9 Max Planck Institute for the Science of Human History, Department of Archaeology, Jena, Germany; 10 Kaziemierz Wielki University, Institute of Geography, Bydgoszcz, Poland; 11 Faculty of Geosciences, Utrecht University, Utrecht, The Netherlands; 12 Polish Academy of Sciences, Institute of Geography and Spatial Organization, Warsaw, Poland; Centre National de la Recherche Scientifique, FRANCE

## Abstract

Fire regime shifts are driven by climate and natural vegetation changes, but can be strongly affected by human land management. Yet, it is poorly known how humans have influenced fire regimes prior to active wildfire suppression. Among the last 250 years, the human contribution to the global increase in fire occurrence during the mid-19^th^ century is especially unclear, as data sources are limited. Here, we test the extent to which forest management has driven fire regime shifts in a temperate forest landscape. We combine multiple fire proxies (macroscopic charcoal and fire-related biomarkers) derived from highly resolved lake sediments (i.e., 3–5 years per sample), and apply a new statistical approach to classify source area- and temperature-specific fire regimes (biomass burnt, fire episodes). We compare these records with independent climate and vegetation reconstructions. We find two prominent fire regime shifts during the 19^th^ and 20^th^ centuries, driven by an adaptive socio-ecological cycle in human forest management. Although individual fire episodes were triggered mainly by arson (as described in historical documents) during dry summers, the biomass burnt increased unintentionally during the mid-19^th^ century due to the plantation of flammable, fast-growing pine tree monocultures needed for industrialization. State forest management reacted with active fire management and suppression during the 20^th^ century. However, pine cover has been increasing since the 1990s and climate projections predict increasingly dry conditions, suggesting a renewed need for adaptations to reduce the increasing fire risk.

## Introduction

Fire has influenced global biogeochemical cycles and natural ecosystems since the late Silurian [1, 2] and has been essential to human evolution since at least the early Pleistocene [1, 3]. Humans have used fire for large-scale land cover control [4–6], which may have affected fire regimes and the atmospheric composition beyond their natural variability over the past several millennia [7–10]. In light of increasing drought occurrence and fire risks due to global climate and land management change [11, 12], it is necessary to consider past climate-human-fire relationships that provide the baseline for current and future adaptation strategies. A key period in shaping modern and future human-fire relations is the 18^th^ and 19^th^ centuries CE [13, 14], when one of the largest socio-ecological transitions in human history—industrialization—significantly altered land use strategies due to rapidly growing population densities and energy demands, with fire becoming less important as a land management tool but rather turned into a threat [3–5].

Global sedimentary charcoal records [15–17] and fire-related CO and CH_4_ concentrations in Antarctic ice cores [18, 19] show that biomass burning peaked during the mid-to-late 19^th^ century and subsequently declined. This increase in fire was mainly attributed to improvement of natural burning conditions at the end of the Little Ice Age (i.e., a warmer, drier climate and increased biomass availability), but also to increased rates of human land-cover change [15, 20–24], with the intentional use of fires to expand grass and agricultural land [25] and in forest management [26]. During the late 19^th^ to early 20^th^ century, both fire occurrence and the area burnt strongly decreased in industrialized areas independent of spatial scale; this is generally attributed to fire suppression due to the reduced importance of fire for human livelihoods [5, 22, 27]. The initiation of fire suppression is mainly associated with thresholds in population densities and landscape fragmentation induced by the expansion of cropland and pastures [14, 28]. Due to fuel accumulation, fire suppression represents a major factor contributing to increasing modern and future fire risks, not only in fire-prone landscapes [29, 30].

Assessment of the reconstructed decadal-scale variability of biomass burning using dynamic vegetation-fire models has revealed a lack in understanding of past fire regimes and emissions [14, 28] for two reasons. First, models based on modern global fire emission data include highly resolved fire regime parameters and burning emission factors [14, 31] that are largely unknown for periods preceding instrumental data [32]. Second, past human—fire—land-use relationships are highly uncertain regarding the relative importance of ignition, suppression, and human impacts on fire regimes, especially during periods predating active fire suppression [14, 33, 34]. These unknowns challenge the capability to reliably predict future fire regime shifts and to adapt to projected increased fire risks.

Guiding future carbon cycle modeling, land management, and nature conservation efforts requires a comprehensive understanding of past fire regimes (i.e., the characteristic frequency, severity, intensity, and seasonality of fire over space and time) combined with information on past (human) land cover and climatic changes [4, 25, 26, 32]. Fire intensity, the rate of energy released per unit fire line (kW m-1) related to burning temperatures and durations, i.e. fire residence time [35], determines combustion efficiency and the severity of impacts on ecosystems, and varies with fuel moisture, rate of spread, and fire type (e.g., surface vs. crown, smoldering vs. flaming fire) [35–37]. Combined with the amount and type of biomass burnt, fire intensity determines the injection height of the smoke plume [38, 39] and absolute emission factors needed to assess the role of fires in biogeochemical cycles [37, 40].

To characterize past fire regimes, fire frequencies and the area and amount of biomass burnt can be reconstructed using sedimentary macrocharcoal (i.e., >150 μm) [41], assuming that larger particles derive from more proximal fires [42–45]. Charcoal, however, provides little information on fire intensities. In atmospheric chemistry, novel molecular markers used to trace biomass burning of low intensities are the monosaccharide anhydrides (MAs) levoglucosan (LVG, 1,6-anhydro-β-D-glucopyranose) and its isomers mannosan (MAN, 1,6-anhydro-β-D-mannopyranose) and galactosan (GAL, 1,6-anhydro-β-D-galactopyranose). These thermal dehydration products of cellulose (LVG) and hemicellulose (MAN, GAL) form at burning temperatures <350°C, thus representing smoldering conditions [46, 47]. Production ratios between MA isomers are mainly related to the type of biomass burnt, i.e., the taxa-specific composition of (hemi-)cellulose [48], burn duration, and the relative contributions of flaming and smoldering phases [49–51]. MAs have shown potential as sedimentary proxies [36, 41, 52–54], because LVG is stable in the atmosphere for several hours to days [55, 56] and is transported attached to aerosols, e.g., charcoal particles [57]. In temperate soils, MA degradation is substantial [58], whereas LVG hardly degrades in the marine water column and only partly in marine surface sediment [59], suggesting that MAs are stable during and after sedimentation in lakes, similar to charcoal [43].

Here, we test the extent to which forest management drove fire activity over the last 250 years. We characterize and quantify source-area specific fire intensities and relative fire sizes as major parameters of fire regimes near an Old-World center of industrialization in the temperate central European lowlands. We use sub-decadal records of macroscopic charcoal (CHAR, in three size fractions) and MAs from the same samples in a varved sediment core of Lake Czechowskie (Tuchola forest, north Poland), spanning 1640–2010 CE, considering age and proxy uncertainties to obtain statistically robust and spatially and temporally explicit fire regime characteristics. Combined with climate information, quantitative land cover reconstructions from pollen data, and analyses of historical maps and documents, we assess the drivers of changing regional fire regimes and put these in context of anthropogenic influences on globally observed fire activity during the 19^th^ century.

## Materials and methods

### Study area and sediment coring

**Fig 1:**
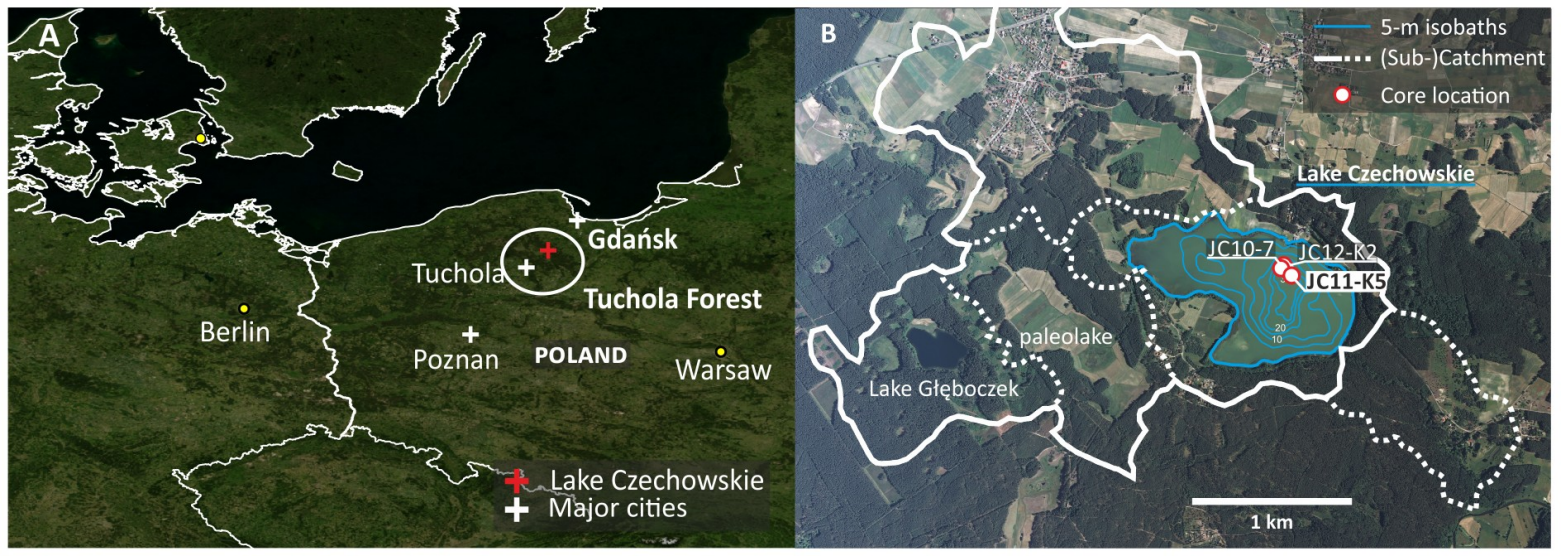
Study area. A) Location of Lake Czechowskie, Tuchola Forest, northern Poland. Map: NASA's Blue marble next generation and state borders by EuroGeographics and UN-FAO. B) The lake catchment, representing the “local scale” referred to in the text, and location of the analyzed sediment core JC11-K5 in the deepest part of the lake. Map: air image provided by provided by Head Office of Geodesy and Cartography, Warsaw, Poland.

The c. 300,000 ha Tuchola forest, north Poland (Fig 1), is characterized by mean annual precipitation and temperature of 570 mm and 7°C during 1951–1980 [60, 61]. Compared to other regions of the world [62], fires are rare and burn small areas (100–250 events per year in Poland, <1 ha per event), occurring mainly during dry summers [63, 64]. Historical documents suggest that a shift in forest management occurred with the first partition of Poland in 1772 CE (Common Era), when northern Poland became Prussian and energy demand for industrialization strongly increased. At the onset of the 18^th^ century, the royal Tuchola forest, as most European forests, was a human-shaped mixed broadleaf forest of reduced carbon stocks [26, 33, 65], due to intensive forest use including charcoal production and fire use to promote heather for beekeeping [66–68]. Yet, a royal decree in 1778 CE and a cabinet order in 1782 CE prohibited the use of fire in forests [69], because forests became main resources for construction wood [67] and state foresters restructured most of the Tuchola forest by planting pine monocultures [26, 69].

Today, c. 90% of the Tuchola forest is covered by single-species, single-aged Scots pine (*Pinus sylvestris*) forest stands with dispersed cropland and pastures [70]. The 77 ha, 32 m deep Lake Czechowskie (53°52′27″N 18°14′12″E, 109 m a.s.l., Fig 1) is located in the northern Tuchola forest in formerly Prussian territory with a historically important route passing north of the lake. The lake’s 1970 ha catchment is composed of glacial till and sandy outwash deposits that limit surface runoff and erosion [26, 71, 72].

**Fig 2:**
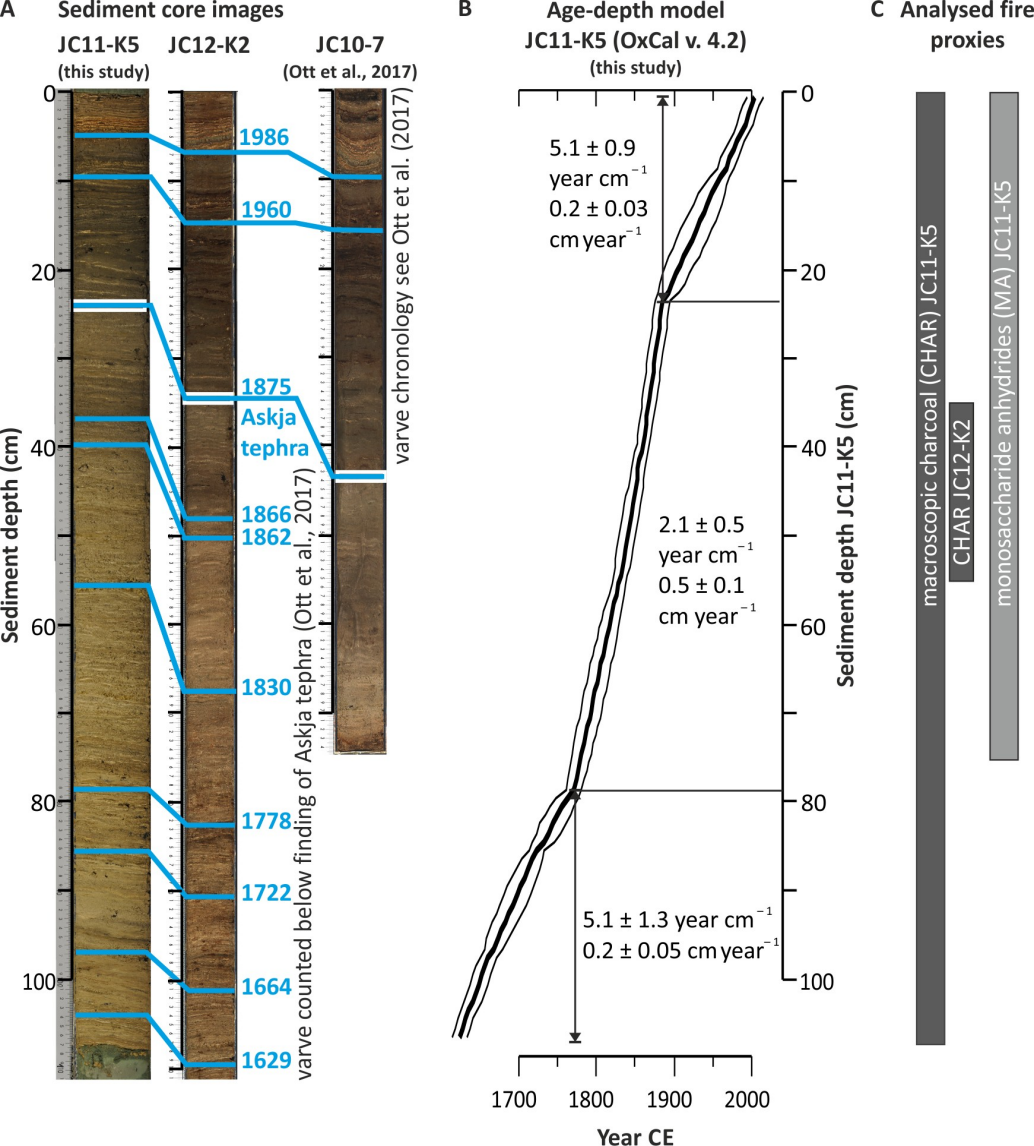
Dating of short core JC11-K5 of Lake Czechowskie. A) Correlation of marker layers (blue) detected in the core image and in short core JC12-K2 (this study) and the core of the master sequence JC10-7 [26, 72]. B) Age-depth model and major changes in sedimentation rates. C) Core sections analyzed for sedimentary charcoal and fire biomarkers.

The sediment core JC11-K5 was recovered in 2011 in 30 m water depth using an UWITEC gravity corer (Fig 1*B*). Sediments were composed of yellowish-brownish organic and calcareous muds that were finely laminated with dry bulk densities and TOC contents of 0.19 ± 0.03 g cm^–3^ and 7.6 ± 1.3% (μ±σ), respectively. Laminations represent calcite varves interrupted by two faintly varved intervals during the mid-20th century, allowing high-resolution reconstruction [72]. JC11-K5 was dated by correlating ten macroscopically visible layers with counted annual layer sequences of adjacent cores (Fig 2). Varve counting of JC12-K2 was performed below the depth of tephra shards at 33 cm related to the Askja eruption in 1875 CE (Fig 2*A*). As a conservative estimate, we assigned a 2σ error of 10 years to the marker layers that we used for calculating the age-depth model in OxCal v. 4.2, a Bayesian age-depth modelling approach that provides posterior age uncertainties [73]. Prominent shifts in sedimentation rates occurred in c. 1770 and 1890 (Fig 2*B*) with higher rates related to higher in-lake productivity (thicker diatom layers, such as the marker layer of 1830 CE) and reworking of littoral material (observations from thin sections; F. Ott, unpublished).

### Multi-(fire) proxy analyses

For sedimentary macroscopic charcoal analysis, 1 cm^3^ of wet sediment was dissolved in water, sieved through a 150-μm mesh. Under a stereomicroscope, macroscopic charcoal of three size classes (150–300, 300–500, and ≥500 μm) was counted continuously throughout the core (*n* = 106, 1630–2011 CE, Fig 2*C*) assuming the largest charcoal particles to represent flaming fires with nearby source areas [43, 44, 74]. To estimate a proxy error that combines sampling, preparation and macrocharcoal counting uncertainties, we continuously sampled short core JC11-K2 between 35–55 cm core depth (n = 20, Fig 2*C*), i.e., interval 1840–1875 CE, that could be linked to core JC11-K5 by four marker layers as determined from varve counting. Samples were processed in the same way as for JC11-K5. The numbers of absolute particles cm^–3^ were compared with the JC11-K5 samples of the same time interval (n = 31) to determine an overall mean relative standard deviation of 0.8% (RSD = 100* σ/μ of each sample for all size classes).

To account for low-intensity fires [46], the topmost 75 samples (1780–2010 CE) were also analyzed for MAs (*n* = 75, 1780–2011 CE, Fig 2*C*): 125–250 mg dry sediment were extracted with a DIONEX Accelerated Solvent Extractor (ASE 200, 100°C, 7.6×10^6^ Pa) using a 9:1 solvent mixture of dichloromethane (DCM):methanol (MeOH). As an internal standard, 2.5–5 ng deuterated levoglucosan (dLVG) was added. The total lipid extracts were separated on an unactivated SiO_2_ gel column (Merck Si60, grade 7754) using sequential elution with DCM:MeOH (9:1) and DCM:MeOH (1:1). The 1:1 fractions were re-dissolved in 95:5 acetonitrile:H_2_O and filtered using a 0.45 μm polytetrafluoroethylene filter before analysis. The MAs were analyzed by ultra-high pressure liquid chromatography-high resolution mass spectrometry using a method adapted from an earlier HPLC-ESI/MS^2^ method [75]. Authentic standards for LVG, GAL and MAN were obtained from Sigma Aldrich, and that for dLVG (C_6_H_3_D_7_O_5_) from Cambridge Isotope Laboratories, Inc. Integrations were performed on mass chromatograms within 3 ppm mass accuracy. Concentrations were corrected for relative response factors to dLVG of 0.997, 0.822, and 2.137 for LVG, MAN, and GAL, respectively. Instrumental (standard) errors for LVG, MAN, and GAL were 4 ± 3, 14 ± 15, and 28 ± 38% (1σ), respectively.

Quantitative land cover estimates were derived from pollen records of varve-dated sediment core JC10-7 in 2-cm steps, i.e., at a resolution of ~5 years/sample [26]. To convert % pollen to land cover, we used the REVEALSinR function of the DISQOVER R package with pollen productivity estimates from the PPE.MV2015 data set and the LSM dispersal model [76].

### Robust proxy records considering age and proxy uncertainties

We provide a robust Monte Carlo based procedure, which adds uncertainty estimates to the existing charcoal record analysis presented by Blarquez, Girardin [77]. The approach starts with influx calculations of CHAR (particles cm^–2^ a^–1^) and MAs (ng cm^–2^ a^–1^), which were derived from a Markov chain Monte Carlo routine that we developed in R version 3.4.4 using the base R functions of the stats package (S1 Code, S1 Fig). Sample age ranges are described by a Gaussian function using μ_age_ and σ_age_ of each depth from the marker layer-based OxCal age-depth model. We randomly calculated 10,000 stratigraphically consistent, positive unit deposition time values for each sample (UDT) to retrieve μ_UDT_ and σ_UDT_ of the UDT distribution by UDT (a cm^–1^) = Δt (a) / Δd (cm) (S1 Code, S1 Fig)

Proxy ranges for each sample are also described by a Gaussian distribution function (μ_proxy_, σ_proxy_ from parallel measurements) to randomly generate *n* normally distributed proxy values (PV). These were divided by *n* randomly generated UDT values (using μ_UDT_ and σ_UDT_) to yield *n* flux values: Flux (proxy unit cm^–2^ a^–1^) = PV (proxy unit) / UDT (a cm^–1^). For the flux density function (pdf_flux_, defined by μ_flux_ and σ_flux_), we multiplied MA values (ng g^–1^) by the sample’s dry bulk density (g cm^–3^), excluding extreme values (i.e., values above the 0.99 quantile) that result from combining exceptionally high PVs with exceptionally low UDTs.

**Fig 3:**
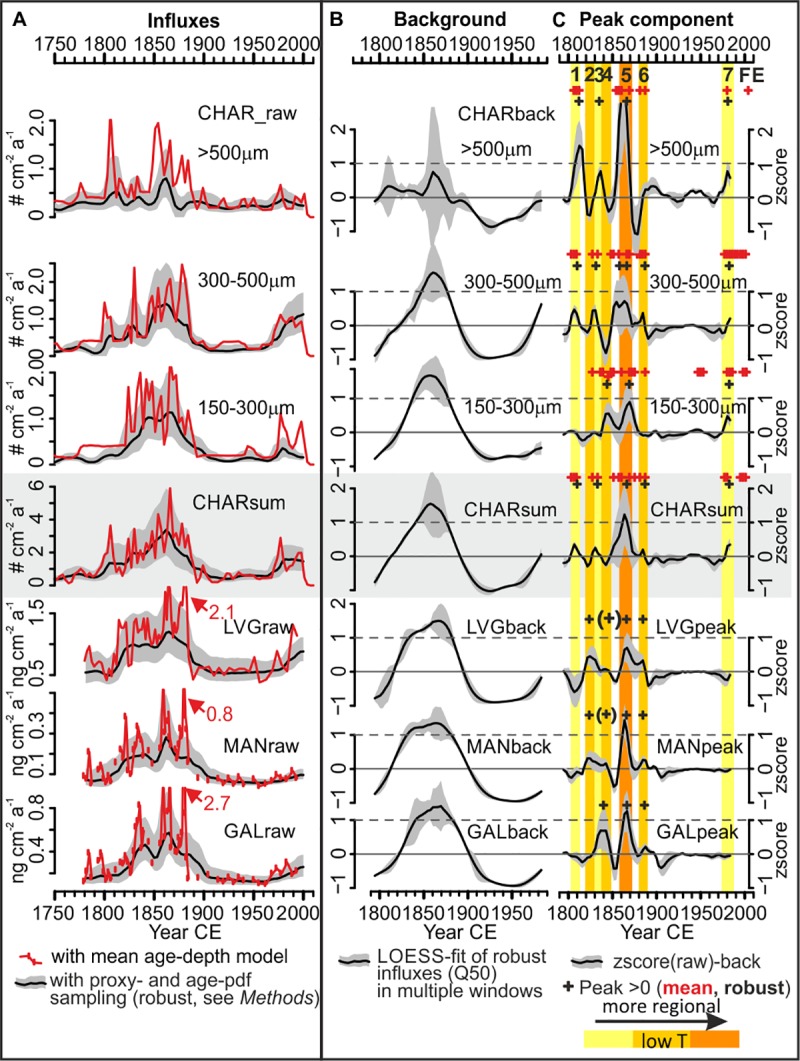
Fire proxy records of Lake Czechowskie, northern Poland. A) Raw macrocharcoal (CHAR, n = 82) and MA (LVG, MAN, GAL, n = 75) influx records. CHAR_sum_ is the summed record of all charcoal particles >150 μm. Black lines and gray polygons are medians and interquartile ranges of robust influx calculations, respectively (*Methods)*. Influxes calculated using the classical mean age-depth model are in red. B) Fire proxy background component. Black lines and gray polygons are medians and Q10–Q90 ranges, respectively, of 1,000 random LOESS fits of the standardized median of the robust influx records (black lines in A) with varying window widths. C) Fire proxy peak components. Black lines and gray polygons are medians and Q10–Q90 ranges, respectively, from subtracting the LOESS-fits of B from the standardized median records of A (black lines). Crosses and colored shaded areas (yellow to orange) mark major positive peaks indicating source area- and temperature-specific fire episodes (FEs1–7, Table 1). Black crosses in brackets mark tentative peaks that were above average only for some window widths. Red crosses mark peaks from decomposition of the mean influx record.

**Fig 4:**
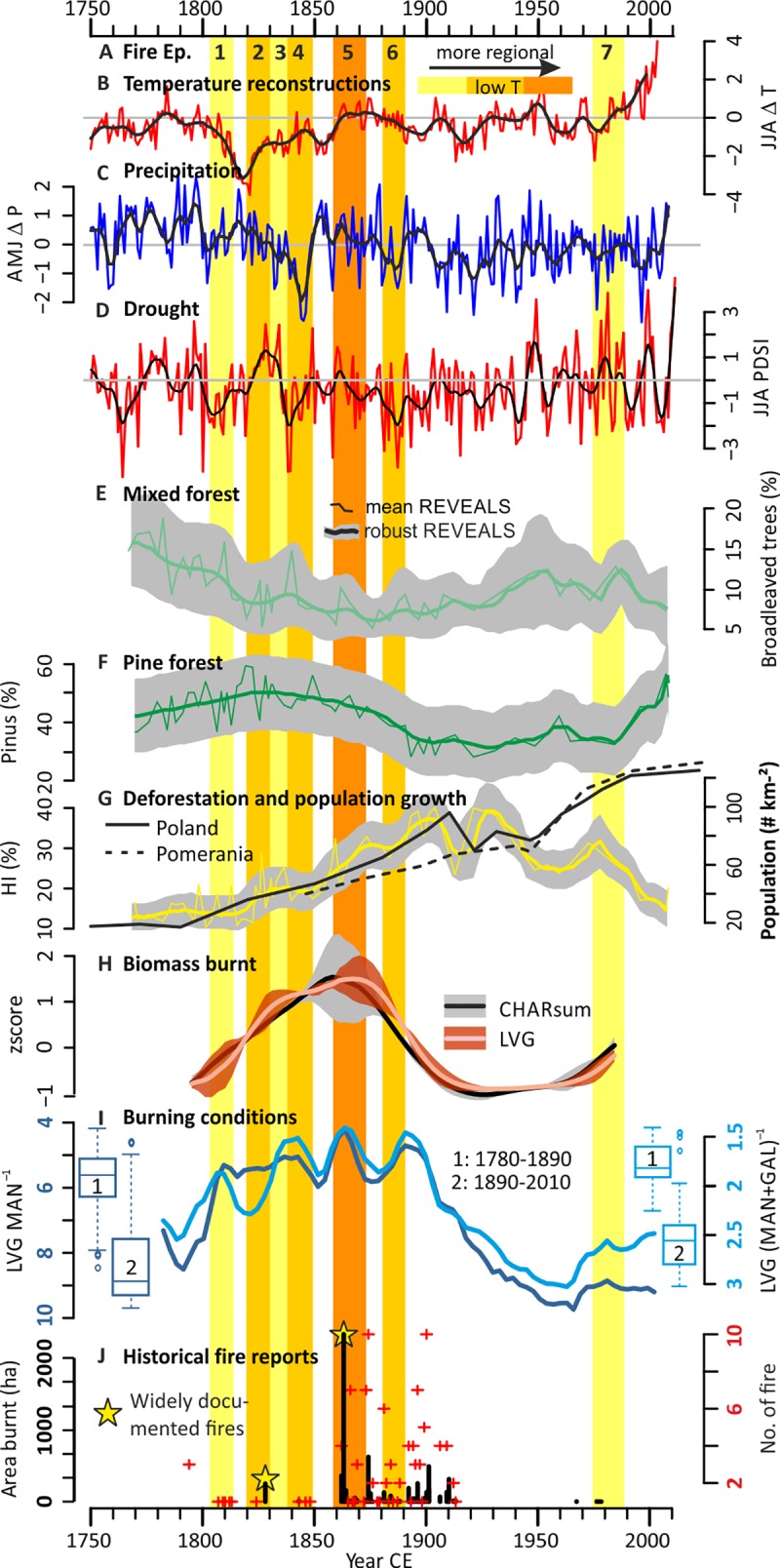
Comparison of fire proxy records with climate, land cover, and historical data. A) Source- and intensity-specific fire episodes (shaded areas from Fig 3C, Table 1); B–C) annual and 20 point LOESS-smoothed June-July-August mean temperatures (JJA Δ T) and April-May-June precipitation (AMJ Δ P) relative to the period 1901–2000 CE [91]. D) Reconstructed Palmer Drought Severity Index (JJA PDSI), reflecting spring-summer soil moisture conditions [92], averaged over the Tuchola area (53.4–54.4°N, 17.3–18.85°E, S2 Fig). E–G) REVEALS-transformed [76] pollen records of the sum of broadleaved taxa (light green), Scots pine (*Pinus sylvestris*, dark green), and human-indicator (HI) taxa (yellow, compared to population densities) from core JC10-7 [26], respectively. Thick lines and gray polygons are medians and Q10–Q90 ranges of the Markov chain Monte Carlo approach (*Methods)*, thin lines are calculated using the classical mean age-depth model. H) Background components of levoglucosan (LVG) and CHAR (CHAR_sum_) from Fig 3B, representing the relative amount of biomass burnt. I) MA ratios representing relative burning conditions (y-axes reversed). J) Minimum estimates of area burnt (ha, black bars) and fire occurrence (red crosses) as reported in historical documents of the Tuchola forest [26] (for 20^th^ century instrumental data see S2 Fig).

To consider the full age uncertainty of a sample, we generated the age density functions pdf_age_ for each sample by combining normalized segments of i) the older tail of the OxCal age distribution for the lower sample boundary, ii) the younger distribution tail for the upper sample boundary, and iii) uniform values between these tails (S1 Code, S1 Fig). Both, pdf_age_ and pdf_flux_ were sampled to generate *n* likely ages and fluxes per sample (S1 Code). Fluxes that fell into evenly spaced 3-year age bins (i.e., median record resolution, S1 Fig) were used to calculate the output statistics (used in Figs 3 and 4*A*).

**Table 1:** Classification of robust peaks in fire proxies in relation to fire regime parameters.

Fire intensity	Fire size	Source area	CHAR	CHAR	CHAR	CHAR	Levo-glucosan	Manno-san	Galacto-san
			150–300	300–500	>500 μm	sum			
Low-High	Large	Regional	1	1	1	1	1	1	1
High	Small-Medium	Local	1	1	3	3	0	0	0
Low	Medium-large	Regional	1	1	0	0	2	2	2

The number of peaks during the period 1800–2000 (black crosses in Fig 3C) and colours as in Fig 3A. Levoglucosan, mannosan and galactosan are only produced by low fire intensities (more produced if more area burnt). Bigger charcoal pieces are generally linked to more nearby source areas. See text for references and further discussions.

In addition, mean fluxes were calculated using the pretreatment function in the paleofire R package using the default parameters (9) and the mean OxCal age-depth model of core JC11-K5 (Fig 2*B*, bold line). A comparison showed that robust fluxes were smoothed, but underestimated absolute mean fluxes due to strongly overlapping pdf_age_ of adjacent samples at 1 cm sample resolution. Hence, we averaged the raw proxy and age values of three adjacent samples before robust flux calculation. Median MA flux records were used to calculate MA ratio records (i.e., LVG MAN^–1^ and LVG (MAN+GAL)^–1^) of the same time resolution.

To provide relative estimates of biomass burnt and fire frequencies, fire proxy records were decomposed into a low-frequency background and a high-frequency peak component, a classical approach in sedimentary charcoal analysis [42, 78, 79], which we adopted here also for MA-record analyses. We performed the statistical decomposition in two ways (Fig 3*B* and 3*C*). First, CHAR records of the classical influx calculation using the mean age-depth model were decomposed translating some of the main principles of the CHARanalysis program [42] to R (S1 Code). Briefly, charcoal records were interpolated to a 3-year median sample resolution and CHAR was calculated using the pretreatment function in the paleofire R package using the default parameters (9) and the mean OxCal age-depth model of core JC11-K5 (Fig 2*B*, bold line). A locally-weighted regression smoothing (LOESS) fit with a half window width (hw) of 5% of the entire record length was used to separate the background from the peak component with the R package locfit [80], i.e. Flux_peak_ (proxy unit cm^–2^ a^–1^) = Flux_raw_−Flux_back_ and Flux_back_ (proxy unit cm^–2^ a^–1^) = LOESS (Flux_raw_, hw = 0.05). With a Gaussian mixture model (package mixtools [81]), the signal peaks were classified as fire events if they exceeded the 99^th^ percentile of the noise distribution [82, 83]. We attributed closely spaced peaks (of adjacent years) to the same fire episode.

Second, we calculate statistically robust background and peak components following the suggestion of Blarquez, Girardin (77) to vary the window widths during background calculation. Briefly, we standardized the medians of the robust CHAR and MA influx records to get comparable units and distributions. Then, we use a Monte Carlo approach to fit a LOESS in varying window widths (i.e., 5–25% of the record length, comparable to [77], 1000 times randomly sampled) as background and subtracted the 1000 LOESS fits from the medians as 1000 peak component records. We mark the above-average peaks using the Monte Carlo approach that are fewer peaks compared to those derived from classical decomposition using the mean age model and one window width (black vs. red crosses, Fig 3*C*), the latter classically interpreted as individual fire events considering noise, e.g., related to re-deposition [42, 77].

Here, we assume that fire episodes (FEs) would result in peaks, even when accounting for age and proxy uncertainties, hence, representing periods of multiple fire events that produced sufficiently high influxes of burning residues to be preserved. We use the presence of robust peaks in CHAR and/or MA records (black crosses, Fig 3*C*) to interpret three types of sub-decadal FEs based on the dominant fire intensity, size, and source area of the burning proxies (Table 1). These are then compared with historically documented fires.

For pollen data, we modified the calculation and used the REVEALS-output (μ_REVEALS_ and σ_REVEALS_) to define the Gaussian distribution function pdf_flux_. For the sum of human indicator taxa (HI, i.e. sum of *Plantago lanceolata*, *Ceralia spec*., *Secale spec*., *Rumex acetosella-var*.), we replaced pdf_flux_ by the summed density functions (pdf_sum_) for each sample generated from *n* sums of randomly drawn REVEALS values of each taxa, allowing only sums ≤100% to sustain realistic land cover percentages.

Historical documents and maps of the Tuchola forest were provided by the State Archives Gdańsk, Bydgoszcz and the State Library and Archive of Prussian Cultural Heritage, Berlin. Many documents were lost and fires were reported sporadically without exact areas measured, especially before 1850 [26, 84]. Hence, documented fire occurrences and extents (Fig 4*I* *and* S2 Fig) are minimum estimates, preventing a more quantitative comparison with fire proxy peaks.

## Results and discussion

### Fire regimes during the last two centuries

All fire proxies increase from below average influxes before 1800 CE (e.g., CHAR_sum_: 0.45 particles cm^–2^ a^–1^, LVG: 0.5 ng cm^–2^ a^–1^) to maximum influxes during the 1860s (CHAR_sum_: 3.4 particles cm^–2^ a^–1^, LVG: 1.2 ng cm^–2^ a^–1^), except the largest CHAR fraction (CHAR_>500μm_) that peaks in the early 1800s and during the 1860s (Fig 3*A*). Influxes then declined to low values by the early 20^th^ century (CHAR_sum_: 0.4 particles cm^–2^ a^–1^, LVG: 0.5 ng cm^–2^ a^–1^) and remained low until c. 1970 when CHAR_300–500μm_ and LVG influxes increased again until their later peaks (CHAR_300–500μm_: 0.8 particles cm^–2^ a^–1^, LVG: 0.88 ng cm^–2^ a^–1^) in the 1980s and 2000s, respectively, whereas CHAR_>500μm_, MAN, and GAL remained low (median robust influxes, calculated using the Monte Carlo-based approach, Fig 3*A*).

We find similar decadal-scale background trends for CHAR and MAs (CHAR_back,_ MA_back_, 1780–2010 CE, Fig 3*B*), which we interpret as relative (not absolute) amount of biomass burnt under various burning conditions and under low temperatures, respectively. CHAR_back_ is known to reflect the regional amount of biomass burnt, although partly affected by sediment reworking and catchment erosion [85, 86]. The latter effect is of limited relevance at Lake Czechowskie as the high sedimentation rates are related to internal productivity [72]. Comparison with the sedimentation rate-independent ratios of the three MA isomers (Fig 4I) shows that MA_back_ (i.e., LVG_back_, MAN_back_, and GAL_back_, Fig 3*B*) also reflects relative changes in biomass burnt. The MA_back_ and CHAR_back_ records are inversely correlated with the MA ratios (e.g., LOESS-fitted LVG MAN^–1^ vs. CHAR_sum_back_: r = –0.8, p < 0.001), which are in the range of modern MA emissions and ratios controlled by the type of biomass burnt and burning conditions, i.e. burn duration, and the relative contributions of flaming and smoldering phases [49–51]. The lower MA ratios and their higher variability before 1890 CE than after (boxplots, Fig 4I), with minimum and maximum values during the 1860s and 1960s, respectively (e.g., LVG MAN^–1^: 4.2 vs. 9.6, Fig 4I) suggests that biomass burning conditions changed significantly in the 20^th^ century.

Yet, the differences between MA_back_ and CHAR_back_ trends suggest varying burning conditions on shorter (sub-decadal) timescales. MA_back_ increased from below average toward 1σ above average anomalies for 15 years longer than CHAR_back_ (1830–1885 vs. 1840–1880 CE, respectively, Fig 3*B*) and reached maximum anomalies a decade later than CHAR_back_ (c. 1870 and 1860 CE, respectively, Fig 3*B*), which we attribute to biomass burnt during distinct fire episodes.

Sub-decadal fire episodes (FEs) are marked by distinct peaks in CHAR and/or MA records (black crosses, Fig 3*C*), with all fire proxies showing higher FE frequencies before than after 1890 CE. CHAR peaks are classically used to reconstruct local fires within ~1 km of the deposit [44, 74], but charcoal can also derive from regional fires within few dozen km [38, 87, 88], e.g., crown fires with high injection columns. Given that charcoal forms under various combustion conditions [43, 44] and MAs represent low burning temperatures (<350°C) [46, 47], the appearance of peaks in all fire proxies in the 1860s (Table 1, Fig 3*C*) suggests that during this period fires of all intensities have produced high amounts of residues, probably from local- to regional source areas. Historically, the largest documented fire episode burnt an area of >2300 ha over several parts of the Tuchola forest during August–September 1863 CE within ~25–30 km of Lake Czechowskie (Fig 4*I* and S2*A* Fig*)*. The closest documented individual fire was ~14 km northeast (~1250 ha burnt, S2*A* Fig*)*, probably providing coarser charcoal particles during crown fires with high injection plumes [38].

In addition, comparison of our robust CHAR or MA peaks with historical data [26] suggests the distinction of two further types of FEs (Table 1): local-scale FEs are represented by three peaks occurring in the coarsest and the total CHAR records during the 1800s, 1830s, and c. 1980 CE, which were not visible in the MA records and only partly in the finer CHAR sizes (FEs 1, 3, 7; Fig 3*C*, Table 1). We interpret these episodes as small and local, e.g., catchment-scale (Fig 1*B*), fires that produced limited MAs due to high burning temperatures (Table 1). Such episodes could represent human-induced fires of high intensity with continued fuel supply such as controlled burning of deforestation residues, e.g., after the sale of the lake shore house in the 1980s (Iwiczno Municipality, pers. comm., March 2018).

Low-intensity, regional FEs relate to prominent peaks in the LVG and MAN records during the 1820s that have no equivalent peak in CHAR anomalies, whereas a prominent GAL peak around 1840 CE corresponds to a peak in CHAR_150-300 μm_ (FEs 2, 4; Fig 3*C*). Documented fires of unknown location burnt an area of 250 ha in 1828 CE [89], and fires burnt >10 ha c. 30–40 km southeast of Lake Czechowskie in 1843 CE [26]: these events may be related to the observed MA peaks (Fig 3*C*). In the 1880s, small MA peaks that are partly reflected in CHAR_peak_ records (FE 6, Fig 3*C*, Table 1) suggest low-intensity fires corresponding to a fire c. 30 km south of the lake in 1887 (S2 Fig) or to the fires ignited by flying sparks (<130 ha) reported along the Starogard-Chojnice railway line [26, 90] (S2*D Fig*).

Hence, we can detect low-intensity fire episodes from the sedimentary record and, supported by historical data, specify previously unknown source regions of sedimentary MAs [37, 52–54]. We find that sedimentary MAs derive from a regional source area, within roughly 50 km of the deposit (S2*A Fig*), recording low-intensity surface or wet-fuel fire events that were large (or long) enough to emit sufficient MAs to be recorded as robust peaks.

### Drivers of fire regime shifts

The period 1780–2010 CE is characterized by prominent shifts in fire regimes. Fire episodes and the amount of biomass burnt increased during the early 18^th^ century until the pronounced FE in the 1860s. After this period, the biomass burnt declined until the 1890s towards changed burning conditions and a 70-year-long period without local-to-regional FEs and characterized by below-average biomass burnt. After the 1960s, regional low-intensity fires slightly increased and a local high-intensity FE occurred in the 1980s (Fig 3*B* and 3*C*). These decadal-scale regional fire regime trends in the Tuchola forest parallel the observed global biomass burning pattern [14–17] and could, hence, serve as an example to study climate-human-fire relationships that could have contributed to the global pattern. Comparing our source-specific fire regime records with tree ring-derived climate reconstructions, i.e., central European temperature and precipitation [91] and the regional interpolation of the Palmer Drought Severity Index (PDSI) [92] (Fig 4*B*–4*D*), quantitative vegetation cover reconstructions from REVEALS-transformed pollen records of the same lake (Fig 4*E*–4*G*), and historical documents (Fig 4*J* and S2 Fig*)* enables an integrative discussion of the primary drivers climate, human impacts and associated natural vegetation changes.

Climate reconstructions do not show comparable decadal-scale trends (Fig 4*B*–4*D*) that would explain the observed trends in biomass burnt and burning conditions (Fig 4*I* and 4*H*) with weather and climate only partly explaining fire occurrences and extents here. In temperate forested ecosystems, fires require summer droughts for fuel drying and fire spread [2], which are reported in historical documents [93] and confirmed by PDSI reconstructions for FEs 1, 4, 5 and 6 (Fig 4*A* and 4*D*). However, some sub-decadal-scale FEs, including the most prominent FE (i.e., FE 5) and low-intensity FEs as reconstructed using MAs do not relate to prolonged droughts alone (Fig 4*A*, 4*C and* 4*D*), as also reported by Zumbrunnen, Bugmann [94]. The most prominent droughts during the 1800s, 1840s, and 1880s did not result in the largest fire extents (e.g., 1828 and 1863 CE, Fig 4*D* and 4*J*), or even no FEs during the minima in PDSI during the 20^th^ century (Fig 4*A*, 4*C and* 4*D*). This suggests that other factors affecting fire extents and spread act on different timescales that we cannot resolve with the inherent uncertainties in our proxy records.

Modern observations also show that natural ignition by lightning is limited, as strikes occur at low frequencies of <5 flashes km^–2^ a^–1^ [95]. Instead, the historical data that we have analyzed suggest that fire ignition was primarily human-triggered, but not necessarily fully independent of weather and climate (as in Roos, Zedeño [96]). Arson during drought periods as a way to show anti-institutional resentments and unintentional human ignition were reported repeatedly, for example, for widespread fires “by a nefarious hand” in the summer of 1863 CE [26, 84] or along the Starogard-Chojnice steam railway in the 1880s [84, 97], respectively (S2*D* Fig*)*. Yet, we exclude the intentional use of fire as a human land management tool for three reasons. First, human-indicator taxa from the same lake (HI, i.e., cereals and ruderals, Fig 4G*)*, a proxy for human deforestation, increased two decades after the increases in biomass burning and reached maximum values in the 1930s when biomass burning was already low (Fig 4*G* and 4*H*). Second, historical maps confirm the HI trends showing significant extension of open land in the region after the increase in fire (early 20^th^ century). Third, fire was banned as a land management tool by Prussian authorities by the late 18^th^ century (see above).

Instead, we find a link between fire regimes, Scots pine cover, and human forest management, as previously suggested [26]. Pine cover increased by at least 10% since the late 18^th^ century and until reaching a maximum around 1830 CE, then declined by ~20% until c. 1910 CE. This trend precedes a similar trend in biomass burnt during the 19^th^ century by roughly three decades (Fig 4*F* and 4*H*). Low MA ratios during the 19^th^ century suggest the burning of softwood, e.g., pine [51], whereas high MA ratios in the 20^th^ century (Fig 4*I*, axes reversed) indicate either the burning of hardwoods, grasses and crops, or both mixed with burned brown coal emissions [48, 49, 51]. Yet, high ratios are also produced under more flaming conditions and higher burning speeds [51] more typical of grass fires [98]. The lack of local-to-regional FEs (Fig 4*A*) suggests that 20^th^-century fires probably occurred outside the Tuchola forest. Hence, we suggest that, here, the co-occurrence of high MA ratios and high HI coverage (Fig 4*G and* 4*I*) represents more grassland and crop-residue burning, whereas low ratios suggest pine fires.

**Fig 5:**
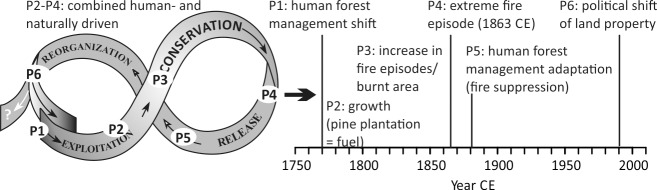
Adaptive cycle of human-induced fire regime shifts during industrialization, including phases P1–6 mentioned in the text (timing for northern Poland). Adapted after Gunderson and Holling [99].

Historical documents suggest that forest management was changed strongly after the 1770s, from mixed broadleaved forests towards pine monocultures in the course of industrialization [26, 69]. We suggest that the state decision to use forests solely as a timber resource initiated an unintended socio-ecological adaptive cycle in forest management strategies (*sensu* Gunderson and Holling [99], Fig 5), superimposed on decadal-scale climate change. Hence, roughly 30 years after the increase in pine cover and decrease of mixed forest (Fig 4*E* and 4*F*), single-aged pine stands with heather (*Calluna vulgaris*) understories [84], i.e. widespread flammable fuel, had grown (phase P2, Fig 5). Supported by some prolonged droughts, biomass burning, fire occurrence and fire hazard were strongly increasing (Fig 4*A* and 4*H*, P3, Fig 5). Compared to broadleaved trees, pine is easily flammable because of its resin-rich needles and its light canopy that results in rapid drying of its understory, even in rather short dry periods in wet years [2, 94, 100]. During the dry summer of 1863, multiple simultaneous fires spread easily in the Tuchola forest [26] (S2A Fig). Hence, the maximum in CHAR and MA records reflects the regional maximum of available and connected fuel that allowed high fire frequencies and extents, even in wetter years (Fig 4*A*, 4*D*, 4*F and* 4*H*, phase P4, Fig 5).

The increased fire risk led to a renewed shift in forest management strategies that included active fire suppression (P5, Fig 5), explaining the reduction in regional FEs and below-average burning since the 1890s (Fig 4*A* and 4*H*). Foresters became firefighters, especially during the early-to-mid-19^th^ century, and arson was an expression of anti-government resentment as historical documents indicate [68, 84]. A planned network of forest tracks to access timber from remote areas [65] was still not in place in 1845 CE (S2*B* and S2*C Fig*). Yet, it appeared as a tighter network after the major FEs in the mid-19^th^ century (S2*D Fig*). The track network increased forest fragmentation and state regulations initiated regular cleaning of forest tracks, which successfully limited fire spread.

Fire occurrence remained low during the 20^th^ century, despite prominent summer droughts as in the 1940s (Fig 4*C and* 4*D*). The expansion of Tuchola’s forest areas from 57% in 1938 CE to 70% in 1990 CE [70] (see also the decline of HI, Fig 4*G*) due to people migrating to expanding cities and abandoning poor soils [70] was dominated by less-flammable broadleaved trees (S2 Fig), probably limiting fire occurrences.

After the 1980s, fire proxy influxes increased again (e.g., LVG, CHAR_300–500μm_, Figs 3 and 2*H*) and MA ratios slightly decreased (i.e., more forest burning, Fig 4*I*), as confirmed by increased instrumentally-measured fire numbers and area burnt in Poland [101] (S3 Fig). HI declined strongly and pine cover increased (Figs 3*A*, 4*F* and 4*G*), which we attribute to changes in land property structures after the end of Communism. Pine monocultures increased on private lands since the 1990s, with >90% of the Tuchola forest being composed of pine today [70]. Together with increasing temperatures across central Europe during recent decades (Fig 4*B*), the fire risk has again increased [26] and possibly requires a renewed adaptation of future forest management (P6, Fig 5).

## Conclusions

Our new approach provides sub-decadal records of sedimentary charcoal and intensity-specific sedimentary fire biomarkers, considering age and proxy measurement uncertainties, to assess the relative importance of specific fire regime parameters in the past (fire intensities, biomass burnt, relative fire extents, burning conditions, and fuel types) that could be included in future data-model comparisons. Compared with land cover and tree ring-based climate reconstructions, we find that since industrialization, human-driven forest management has fundamentally changed human-fire relationships.

Fire was an important land use and land management tool in the central European lowlands and globally since at least Mesolithic, and especially since Neolithic times [4, 10, 25]. The close human-forest and human-fire relationships terminated when fire was replaced by other agricultural measures [3, 5], banned from forests by state authorities, or unintendedly promoted by replacing forest with more flammable taxa, as described here for Poland. Hence, considering not only the conversion from forest to open land with increasing population densities, but also internal forest type conversions could help to improve further dynamic vegetation-fire modelling and comparisons with sedimentary proxy records that should account for several types of uncertainties.

Here, we support previous conclusions [25, 26] that the fire trends during the 19^th^ century, as visible in global and continental charcoal compilations, were primarily influenced by humans, even before active fire suppression, closely linked with and superimposed by natural causes [15, 21, 96]. Sociopolitical shifts during industrialization could have driven unintended adaptive socio-ecological cycles that affected forest composition, fire regimes, and biogeochemical cycles [33, 34]. Timber became a precious resource, not only in Poland, and pine spread far beyond its potential natural distribution [100], similar to other highly flammable pioneer tree monocultures, such as *Eucalyptus spec*. in the subtropics and tropics (i.e., other regions of low natural flammability that were industrializing during the 18^th^ and 19^th^ centuries). Given these preconditions for current and future fire risks and the increased likelihood of summer droughts under future climate change [11, 12], forest management could either invest in further fire suppression measures or, by entering a new adaptive cycle, diversify monocultures to include less-flammable broadleaved taxa to prevent fire spread and further forest disturbances [26, 102, 103].

## Supporting information

S1 Fig Concept of Monte Carlo approach combing proxy and age probability density functions to statistically model robust proxy (influx) values. The Q25 to Q75 range as polygon and the median (Q50) proxy fluxes as lines in the right image.
(TIF)

S2 Fig Regional fires in the Tuchola Forest and road network adaptation. A) Reported locations and extents of fire events in historical documents (State Archive in Gdańsk, compiled in ref. [26]). Map: 2018 OpenStreetMap and contributors, license CC-BY-SA, modified with ArcGIS Desktop: Release 10.2.2. ESRI 2014. Redlands, CA: Environmental Systems Research Institute. B-D) Historical maps with location of Czechowskie catchment (Fig 1*B*) indicating road network within forests: B) planned, manually drawn on the map by Prussian government authorities; C) still historical (pre-industrial) road network and D) realization of planned network (map: For better visibility and example of the tracks in forest were redrawn in pink (denser network in D than planned in B to limit fire spread). Map sources with CC-BY open access license: B) “Karte von den Provinzen Litthaen, Ost- und West-Preussen nebst dem Netzdistrict”, Kart. N 1020, Blatt 92 provided by Staatsbibliothek zu Berlin—Preußischer Kulturbesitz; C) “Topographische Specialkarte des Preussischen Staats und der angrenzenden Länder (Reyman’s Special-Karte)”, signature PAN.C163, sheet 31 and D) “Messtischblatt” signature PAN.C633, sheet 2175; maps of C and D provided by Centralna Biblioteka Geografii I Ochrony Srodowiska IGiPZ PAN.
(TIF)

S3 Fig Total number of fires (bars) and burned area of forests (red line) in Poland in the period 1948–2018. Data from ref. [101], Statistical Yearbook of Forestry, 2018, GUS Statistics Poland, Warsaw and Statistical data of the Polish State Fire Service KG PSP [source: www.kgpsp.gov.pl, last access: 09.08.2019].
(TIF)

S1 Code Basic principles of CharAnalysis and Monte Carlo approach considering combined age and proxy uncertainties. (DOCX)
