# Supplementary figures and images for: Human-induced fire regime shifts during 19th century industrialization: A robust fire regime reconstruction using northern Polish lake sediments
Source: PLoS One. 2019 Sep 16;14(9):e0222011. doi: 10.1371/journal.pone.0222011 (PMC6746370; doi:10.1371/journal.pone.0222011)

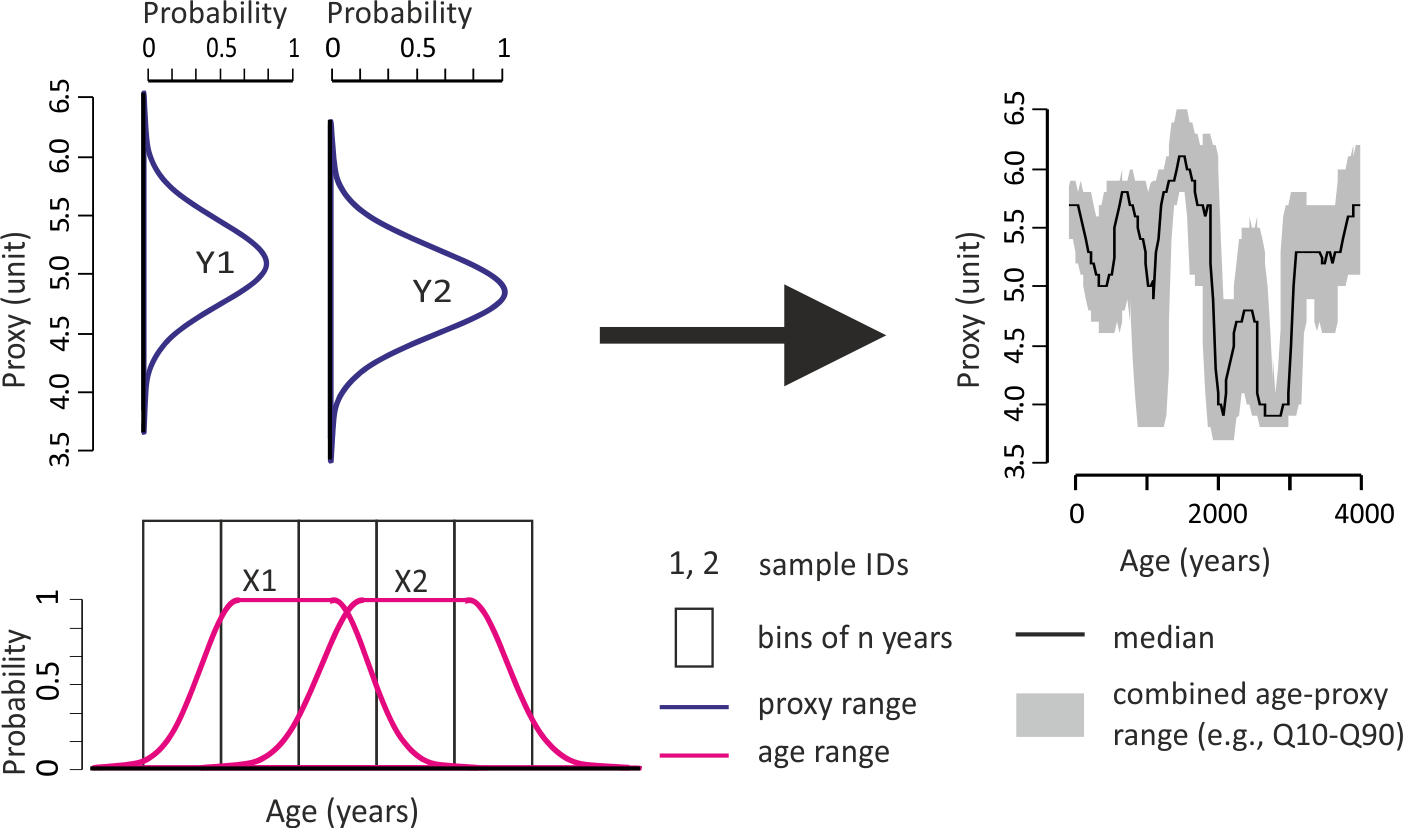

Supplement: S1 Fig — The Q25 to Q75 range as polygon and the median (Q50) proxy fluxes as lines in the right image. (TIF) [file pone.0222011.s001.tif]

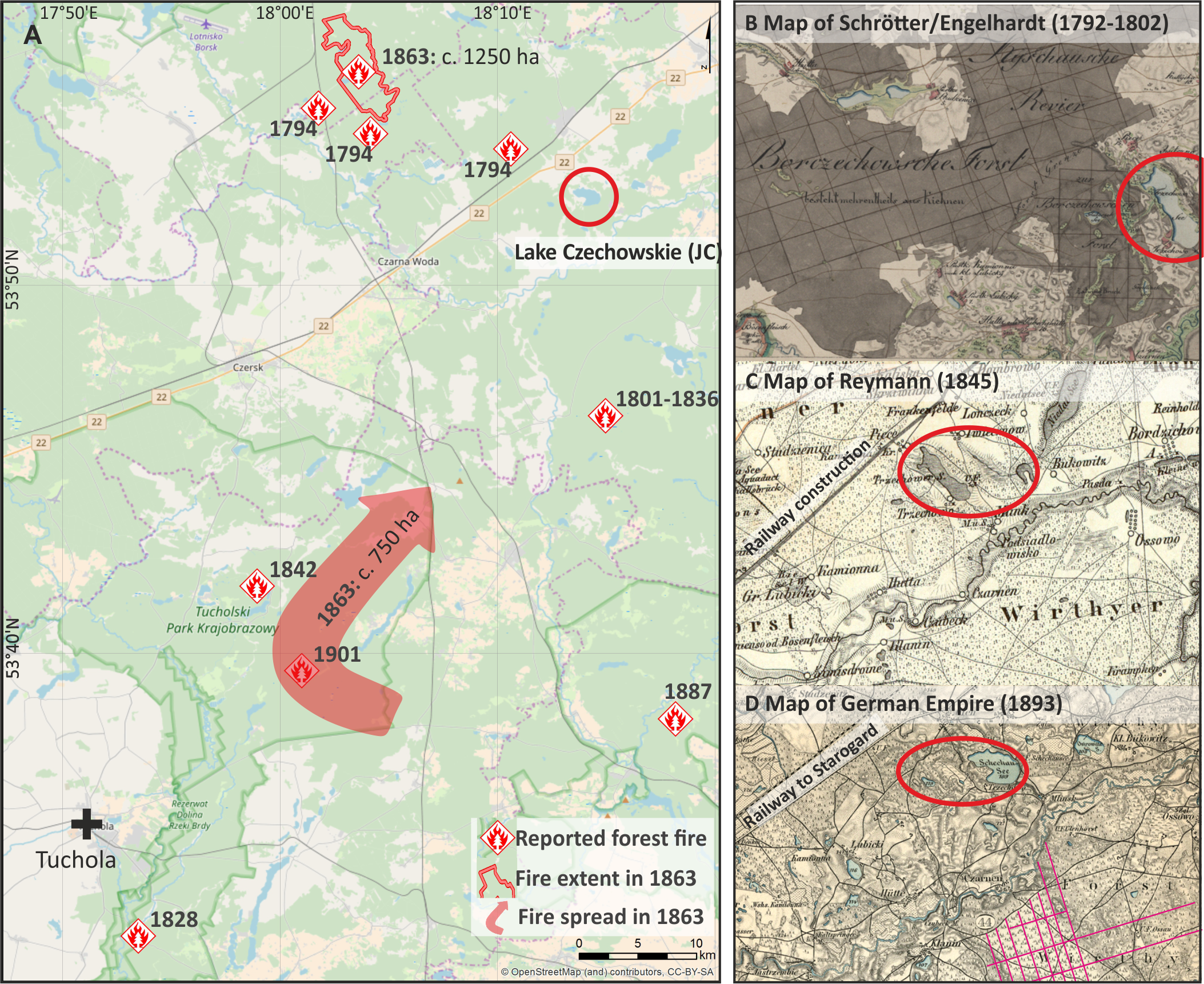

Supplement: S2 Fig — A) Reported locations and extents of fire events in historical documents (State Archive in Gdańsk, compiled in ref. [26]). Map: 2018 OpenStreetMap and contributors, license CC-BY-SA, modified with ArcGIS Desktop: Release 10.2.2. ESRI 2014. Redlands, CA: Environmental Systems Research Institute. B-D) Historical maps with location of Czechowskie catchment (Fig 1B) indicating road network within forests: B) planned, manually drawn on the map by Prussian government authorities; C) still historical (pre-industrial) road network and D) realization of planned network (map: For better visibility and example of the tracks in forest were redrawn in pink (denser network in D than planned in B to limit fire spread). Map sources with CC-BY open access license: B) “Karte von den Provinzen Litthaen, Ost- und West-Preussen nebst dem Netzdistrict”, Kart. N 1020, Blatt 92 provided by Staatsbibliothek zu Berlin—Preußischer Kulturbesitz; C) “Topographische Specialkarte des Preussischen Staats und der angrenzenden Länder (Reyman’s Special-Karte)”, signature PAN.C163, sheet 31 and D) “Messtischblatt” signature PAN.C633, sheet 2175; maps of C and D provided by Centralna Biblioteka Geografii I Ochrony Srodowiska IGiPZ PAN. (TIF) [file pone.0222011.s002.tif]
