# Supplementary material for: Human-induced fire regime shifts during 19th century industrialization: A robust fire regime reconstruction using northern Polish lake sediments
Source: PLoS One. 2019 Sep 16;14(9):e0222011. doi: 10.1371/journal.pone.0222011 (PMC6746370; doi:10.1371/journal.pone.0222011)
Supplement: S1 Code — (DOCX) [file pone.0222011.s004.docx]

S4 Code

Basic principles of CharAnalysis and Monte Carlo approach considering combined age and proxy uncertainties

supplement to Dietze et al. PLOSone

1) Basic principles of CHARanalysis

## Classical charcoal decomposition following CharAnalysis in Matlab after Higuera et al. (2009), i.e. initial translation steps to R @ Olivier Blarquez (2018)

library(locfit)

library(mixtools)

library(paleofire)

df <- data.frame(depthtop=data$depth,

depthbot=c(1:length(data$depth)),

agetop=1950-data$age,

agebot=1950-c(data$age[2:length(data$age)],NA),

volume=1, # in cm3

CHAR=data$proxy)

df <- as.matrix(na.omit(df))

CHAR <- paleofire::pretreatment(df[,1:5],df[,6]) # charcoal influx, using median time resolution (default)

x <- CHAR$ybpI

y <- CHAR$accI # data that interests us i.e. interpolated accumulation rates

# We can smooth that data using for example a lowess

win <- (max(x)-min(x))*0.10 # a rough estimate could be a window width of 10% of the entire record

locbootA <- locfit(y ~ lp(x, deg = 1, h = win/2),

maxk = 500,

family = "qgauss")

predbootA <- predict(locbootA, newdata = x, se.fit = TRUE)

plot(x,y,type="l",lty=2)

lines(x,predbootA$fit)

# Peak component is raw char data minus long term trend

peak <- y-predbootA$fit

peak1 <- peak[peak>0] # consider only positive peaks

plot(peak, type="h")

hist(peak1, breaks=20)

# Gaussian mixture model to differentiate between char fire and char noise, the two constituents of the peak component

gm <- normalmixEM(peak1, k=2)

plot(gm,which=2)

# Usually the treshold is defined as the 99th percentile of the distribution of the noise component

treshold <- gm$mu[1]+qnorm(0.99)*gm$sigma[1];treshold

fire_pos <- which(peak>treshold)

fire_bp <- x[fire_pos]

fire_ad <- 1950-fire_bp #fire dates in AD

# In the charanalysis software there is an additional Poisson test for excluding charcoal peaks coming from the same distribution (i.e. poisson distribution), not included here

plot(1950-x,y,type="l",lty=1, xlim =c(1760, 2012))

lines(1950-x,predbootA$fit,type="l", col = 3, lwd=2)

plot(1950-x,peak, type="h",xlim = c(1760, 2012))

points(1950-x[fire_pos], peak[fire_pos],pch="+",col=3, lwd=2)

2) Monte Carlo approach: considering combined age and proxy uncertainties

R function “model_flux”

This function models the uncertainty imposed on a proxy data set with continuously sampled proxy values and their respective proxy error in stratigraphic order. Depth (cm) and age (+ respective error) should mark the upper and lower boundary of a sample (not the center point). The approach consists of several successive steps. First, a density estimate of the proxy flux (i.e., reciprocal of sedimentation rate) is calculated based on Monte Carlo simulations. Then an age density is calculated by combining the mean ages and sd of ages of upper and lower boundary of a samples. Bins of a certain time resolution are created and in a combined MC run, the density distributions of the fluxes are randomly sampled from all samples that have an age probability in this age bin. Finally, the resulting distributions per bins are characterized by median and interquartile ranges.

#' @param data \code{Data frame}, input data set. Must contain the following data in the specified order: \code{depth (cm)}, \code{age (a)}, \code{age uncertainty (a)}, \code{proxy value} and #' \code{proxy uncertainty}. Note that age is not calendar years but years before present.

#'

#' @param n \code{Numeric} value, number of Monte Carlo runs. Default is \code{100}.

#'

#' @param resolution_density \code{Numeric} value, temporal resolution of the' density estimates during calculation. Default is \code{0.1} years.

#'

#' @param resolution_out \code{Numeric} value, temporal resolution of the output data set. Default is \code(5) years.

#'

#' @param n_density \code{Numeric} value, number of values for density estimates. Default is \code{5000}.

#'

#' @param scale \code(6) value, option to scale (z-transform the data), default is \code{FALSE}.

#'

#' @param k \code{Numeric} vector of length two. Smoothing factors for the plot option \code{"probability"}. First value defines smoothing in x-direction, second smoothing in y-direction. By default, smoothing is only performed in y-direction, i.e., \code{k = c(0, n)}, where n is set to 1 % of n_density.

#'

#' @param quantiles \code{Numeric} vector of length three, quantiles to be used for output. Default is \code{c(0.25, 0.50, 0.75)}.

#'

#' @param plot \code{Character} value, optional keyword to define type of plot output. One out of \code{"quantiles"}, \code{"probability"} and \code{"none"}. Default is \code{"probability"}.

#'

#' @param \dots Further arguments passed to the plot output

model_flux <- function(

data,

n = 100,

resolution_density = 0.1,

resolution_out = 3,

n_density = 5000,

scale = FALSE,

k,

quantiles = c(0.1,0.25, 0.50, 0.75,0.9),

plot = "probability",

...

) {

## check input data

if(sum(names(data) == c("depth",

"age",

"age_error",

"proxy",

"proxy_error")) < 5) {

stop("Input data structure is not correct! See function documentation.")

}

## PART 1 - Generate unit deposition time -----------------------------------

t_unit_raw <- lapply(X = 1:n, FUN = function(i, data) {

## draw random age estimate

age_i <- stats::rnorm(n = nrow(data),

mean = data$age,

sd = data$age_error)

## calculate age differences

age_diff <- c(NA,diff(age_i))

## set inverted ages to NA

age_diff[age_diff < 0] <- NA

## calculate unit deposition time

t_unit <- age_diff / diff(c(NA, data$depth))

## return result

return(list(t_unit = t_unit,

age = age_i))

},

data = data)

## convert list to matrices

t_unit <- do.call(cbind, lapply(X = t_unit_raw, FUN = function(X) {X$t_unit

}))

t_age <- do.call(cbind, lapply(X = t_unit_raw, FUN = function(X) {X$age

}))

## calculate mean unit times

t_unit_mean <- apply(X = t_unit, MARGIN = 1, FUN = mean, na.rm = TRUE)

## calculate sd sedimentation rate

t_unit_sd <- apply(X = t_unit, MARGIN = 1, FUN = sd, na.rm = TRUE)

flux_raw <-

lapply(X = 1:n, FUN = function(X, data, t_unit_mean, t_unit_sd) {

## draw random sedimentation rate estimates

sed_i <- stats::rnorm(n = nrow(data) - 1,

mean = t_unit_mean[2:nrow(data)],

sd = t_unit_sd[2:nrow(data)])

## identify NAs

i_na <- is.na(data$proxy[-1])

## replace NAs by zero

data_0 <- data

data_0[i_na, 4:5] <- 0

proxy_i <- stats::rnorm(n = nrow(data_0) - 1,

mean = data_0$proxy,

sd = data_0$proxy_error)

## replace zero data by NA

proxy_i[i_na] <- NA

## calculate age differences

proxy_flux <- proxy_i / sed_i

## set inverted ages to NA

proxy_flux[proxy_flux < 0] <- NA

## return result

return(proxy_flux)

},

data = data,

t_unit_mean = t_unit_mean,

t_unit_sd = t_unit_sd)

## convert list to matrix

flux <- do.call(cbind, flux_raw)

## calculate empiric density function

flux_density <- apply(X = flux, MARGIN = 1, FUN = function(x) {

d <- try(stats::density(x = x, na.rm = TRUE,

from = 0,

to = 1.1 * max(x, na.rm = TRUE),

n = n_density),

silent = TRUE)

if(class(d) == "try-error") {

d <- NA

}

return(d)

})

data_order <- data[order(data$age),]

da_min <- data_order$age_error[data_order$age == min(data_order$age)]

da_max <- data_order$age_error[data_order$age == max(data_order$age)]

## convert age and age uncertainty to pairwise list

age_info <- as.list(as.data.frame(rbind(data_order$age,

data_order$age_error)))

## define age density age vector

age_index <- seq(from = min(data_order$age) - 5 * da_min,

to = max(data_order$age) + 5 * da_max,

by = resolution_density)

## calculate densities for each age value

density_raw <- lapply(X = age_info, FUN = function(age_info, age_index) {

stats::dnorm(x = age_index, mean = age_info[1], sd = age_info[2])},

age_index = age_index)

ii <- seq(from = 1, to = length(age_index))

## generate output data set

age_density <- vector(mode = "list",

length = length(age_info) - 1)

## generate all density estimates

for(i in 1:(length(age_info) - 1)) {

i_l <- ii[density_raw[[i]] == max(density_raw[[i]])][1]

i_u <- ii[density_raw[[i + 1]] == max(density_raw[[i + 1]])][1]

## define lower tail age vector

d_l <- density_raw[[i]][1:i_l]

## define upper tail age vector

d_u <- density_raw[[i + 1]][i_u:length(ii)]

##normalise density vectors

d_l_n <- d_l / max(d_l)

d_u_n <- d_u / max(d_u)

## define central part density vector

d_m <- rep(1, i_u - i_l -1)

## merge age and density vector

d_merged <- c(d_l_n, d_m, d_u_n)

## normalise merged density vector

d_merged_n <- d_merged / sum(d_merged)

## generate and assign output data set

age_density[[i]] <- d_merged_n

}

## define output object

flux_combined <- vector(mode = "list",

length = length(age_density))

for(i in 1:length(age_density)) {

age <- sample(x = age_index,

size = n,

replace = TRUE,

prob = age_density[[i]])

if(is.na(data$proxy[i + 1])) {

proxy <- rep(NA, times = n)

} else {

if(scale == TRUE) {

flux_density[[i]]$x <- scale(flux_density[[i]]$x)

}

if(is.na(flux_density[[i]][1]) == FALSE) {

proxy <- sample(x = flux_density[[i]]$x,

size = n,

replace = TRUE,

prob = flux_density[[i]]$y)

} else {

proxy <- rep(NA, n)

}

}

flux_combined[[i]] <- data.frame(age = age,

proxy = proxy)

}

x_raw <- unlist(lapply(X = flux_combined,

FUN = function(x) x$age))

y_raw <- unlist(lapply(X = flux_combined,

FUN = function(x) x$proxy))

bins <- seq(from = min(data$age),

to = max(data$age),

by = resolution_out)

data_out <- matrix(nrow = length(bins) - 1, ncol = 3)

density_out <- vector(mode = "list",length = length(bins) - 1)

colnames(data_out) <- paste("q_", quantiles, sep = "")

density_global <- density(x = y_raw, n = n_density,

from = min(y_raw),

to = quantile(y_raw, 0.999),

na.rm = TRUE)

for(i in 1:(length(bins) - 1)) {

x_i <- y_raw[x_raw > bins[i] & x_raw <= bins[i + 1]]

data_out[i,] <- stats::quantile(x = x_i, probs = quantiles, na.rm = TRUE)

density_out[[i]] <- try(density(x = x_i, n = n_density, bw = density_global$bw,

from = min(density_global$x),

to = max(density_global$x),

na.rm = TRUE),

silent = TRUE)

if(class(density_out[[i]]) == "try-error") {

density_out[[i]] <- data.frame(x = NA,

y = rep(NA, n_density))

}

}

extraArgs <- list(...)

if ("main" %in% names(extraArgs)) {

main <- extraArgs$main

}

else {

main <- ""

}

if ("xlab" %in% names(extraArgs)) {

xlab <- extraArgs$xlab

}

else {

xlab <- "Time"

}

if ("ylab" %in% names(extraArgs)) {

ylab <- extraArgs$ylab

}

else {

ylab <- "Proxy value"

}

if ("xlim" %in% names(extraArgs)) {

xlim <- extraArgs$xlim

}

else {

xlim <- range(bins[-1])

}

if ("ylim" %in% names(extraArgs)) {

ylim <- extraArgs$ylim

}

else {

ylim <- range(data_out, na.rm = TRUE)

}

if(plot == "quantiles") {

if ("col" %in% names(extraArgs)) {

col <- extraArgs$col

}

else {

col <- "black"

}

## create empty plot

plot(NA,

xlim = xlim,

ylim = ylim,

xlab = xlab,

ylab = ylab,

main = main)

## divide data set to NA-free snippets

i_na <- c(diff(is.na(rowSums(data_out))), 0)

i_from <- c(1, seq(from = 1, to = length(i_na))[i_na == -1] + 1)

i_to <- seq(from = 1, to = length(i_na))[i_na == 1]

if(length(i_to) == 0) {i_to <- length(i_na)}

if(length(i_to) < length(i_from)) {i_to <- c(i_to, length(i_na))}

bins_plot <- bins[-1]

for(i in 1:length(i_from)) {

graphics:: polygon(x = c(bins_plot[i_from[i]:i_to[i]],

rev(bins_plot[i_from[i]:i_to[i]])),

y = c(data_out[i_from[i]:i_to[i],1],

rev(data_out[i_from[i]:i_to[i],3])),

col = adjustcolor(col = col, alpha.f = 0.3),

border = NA)

graphics::lines(x = bins_plot[i_from[i]:i_to[i]],

y = data_out[i_from[i]:i_to[i],2],

col = col)

}

} else if(plot == "probability") {

if ("col" %in% names(extraArgs)) {

col <- extraArgs$col

}

else {

col <- colorRampPalette(colors = c("white", "black"))

}

## convert density estimates to plot matrix

density_plot <- do.call(rbind, lapply(X = density_out, FUN = function(x) {

x$y

}))

## get bins with NAs

bin_na <- is.na(rowSums(density_plot))

## get/set smoothing factors

if(missing(k) == TRUE) {

k <- c(1, 0.01 * n_density)

}

## average density plot matrix

density_plot <- t(apply(X = density_plot,

MARGIN = 1,

FUN = caTools::runmean,

k = k[2]))

## average density plot matrix

density_plot <- apply(X = density_plot,

MARGIN = 2,

FUN = caTools::runmean,

k = k[1])

## re-inset NAs

density_plot[bin_na,] <- rep(NA, ncol(density_plot))

## generate image plot

graphics::image(x = bins[-1],

y = density_global$x,

z = density_plot,

col = col(n = 500),

xlim = xlim,

ylim = ylim,

xlab = xlab,

ylab = ylab,

main = main)

}

data_out <- list(summary = data.frame(t_lower = bins[-length(bins)],

t_upper = bins[-1],

q_25 = data_out[,1],

q_50 = data_out[,2],

q_75 = data_out[,3]),

data = flux_combined,

densities = density_out)

return(data_out)

}
